# Supplementary material for: Shining light on the microscopic resonant mechanism responsible for cavity-mediated chemical reactivity
Source: Nat Commun. 2022 Dec 19;13:7817. doi: 10.1038/s41467-022-35363-6 (PMC9763331; doi:10.1038/s41467-022-35363-6)
Supplement: Supplementary file 1 — Supplementary Information [file 41467_2022_35363_MOESM1_ESM.pdf]

Supplementary Information Shining Light on the Microscopic Resonant Mechanism Responsible for Cavity-Mediated Chemical Reactivity

Christian Schäfer,[1,2,3,4,*] Johannes Flick,[5,6,7,8,†] Enrico Ronca,[9,‡] Prineha Narang,[6,§] and Angel Rubio[1,2,5,¶]

[1] *Max Planck Institute for the Structure and Dynamics of Matter and Center for Free-Electron Laser Science & Department of Physics, Luruper Chaussee 149, 22761 Hamburg, Germany*
[2] *The Hamburg Center for Ultrafast Imaging, Luruper Chaussee 149, 22761 Hamburg, Germany*
[3] *Department of Physics, Chalmers University of Technology, 412 96 Göteborg, Sweden*
[4] *Department of Microtechnology and Nanoscience, MC2, Chalmers University of Technology, 412 96 Göteborg, Sweden*
[5] *Center for Computational Quantum Physics, Flatiron Institute, 162 5th Ave., New York, 10010 NY, USA*
[6] *John A. Paulson School of Engineering and Applied Sciences, Harvard University, Cambridge, Massachusetts 02138, USA*
[7] *Department of Physics, City College of New York, New York, New York 10031, USA*
[8] *Department of Physics, The Graduate Center, City University of New York, New York, New York 10016, USA*
[9] *Istituto per i Processi Chimico Fisici del CNR (IPCF-CNR), Via G. Moruzzi, 1, 56124, Pisa, Italy*

(Dated: November 27, 2022)

I. SUPPLEMENTARY METHODS

Supplementary Figure 1 illustrates the vibrational spectrum including the PTA+F^- initial state and an intermediate strongly stretched state. During the evolution of the reaction, the vibrational modes are constantly changing which allows many more frequencies to dynamically couple to the cavity than the equilibrium PTA or PTAF^- structures would suggest. The dynamic shift of the vibrational mode during the reaction could contribute to broadened resonances.

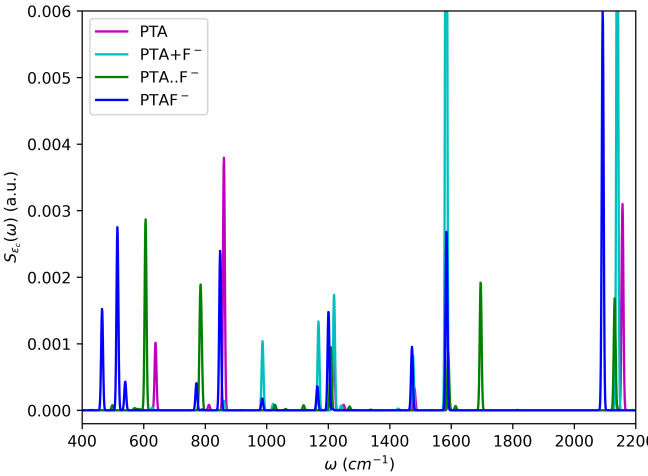

Supplementary Figure 1. Vibrational absorption spectrum along the cavity polarization direction $S_{\varepsilon_c}(\omega) = 2\omega \sum_{j=1}^{N_{vib}} |\boldsymbol{\varepsilon}_c \cdot \mathbf{R}(\omega_j)|^2 \delta(\omega - \omega_j)$ for the bare PTA complex (magenta), our initial state PTA+F^- (cyan), an intermediate state with strongly stretched Si-F bond of 2.889 Å (green), and the pentavalent PTAF^- complex (blue). Vibrational resonances are artificially broadened. In agreement with fig. 1 (d), the polarization of the coupled cavity mode is aligned along the Si-C axis. This linear response calculation used the default normalization implemented in Octopus while all other used a new normalization.

The vibro-polaritonic spectrum in direction of polarization for different light-matter coupling strength is plotted in Supplementary Figure 2. The results are comparable to fig. 1 in the main manuscript but utilize the finite-difference Born-Oppenheimer approach outlined in [1]. For large light-matter coupling (as used in fig. 4), the R^2 self-polarization term (black dashed-dotted) leads to a sizeable blue-shift of the excitation. Comparison with fig. 4 (b) suggests that this effect is responsible for the observed blue-shift with increasing frequency as we keep $g_0/\hbar\omega$ constant. Finite-difference and time-dependent vibrational linear response calculations are slightly shifted

* Electronic address: christian.schaefer.physics@gmail.com
† Electronic address: jflick@flatironinstitute.org
‡ Electronic address: enrico.ronca@pi.ipcf.cnr.it
§ Electronic address: prineha@seas.harvard.edu
¶ Electronic address: angel.rubio@mpsd.mpg.de

with respect to each other.

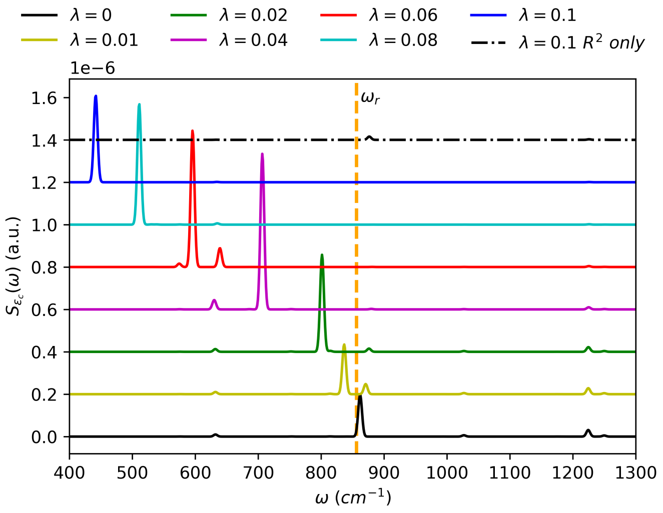

Supplementary Figure 2. Vibro-polaritonic absorption spectrum along the cavity polarization direction $S_{\varepsilon_c}(\omega) = 2\omega \sum_{j=1}^{N_{vib}} |\varepsilon_c \cdot \mathbf{R}(\omega_j)|^2 \delta(\omega - \omega_j)$ for the PTA complex coupled to a cavity mode with increasing coupling strength λ $[a.u.]$. The orange-dashed vertical line indicates the selected cavity frequency. The black-dashed curve represents the PTA spectrum when affected by the self-polarization term of the cavity only, i.e., no bilinear coupling is included. The value of $\lambda = 0.1$ a.u. corresponds to $g_0/\hbar\omega_c = 1.132$.

The here chosen semi-classical treatment of light does not change the ground-state potential energy-surface itself but can merely affect the dynamic of the system. Surely, (ultra-)strong light-matter coupling will lead to correlated ground-states comprising electronic, nuclear and photonic degrees of freedom. It has been shown, that already this path allows strong light-matter coupling to influence electronic structure and thus chemistry [2–7]. In the present work, calculations using the KLI functional [5] suggested very small modifications of the electronic structure when strongly coupled to the cavity.

In order to eliminate the possibility that the solvent is an integral component of the mechanism that is put forward here, we used ORCA with a continuous PCM model description for the solvent methanol. We obtain the energetic values for reactant [PTA+F] = -816.131244 H, pentavalent complex [PTAF]- = -816.1943141865 H, transition-state [PA ... Me3SiF] = -816.18146281 H and product [PA- + Me3SiF] = -816.1846728 H. We obtain an enthalpy between pentavalent complex and transition-state of $\Delta H^\dagger \approx 0.35$ $eV \approx 34$ kJ/mol in good agreement with experimental results (30 and 39 kJ/mol) [8, 9]. Ignoring entropic features as well as bond termination with hydrogen, the resulting product is higher in energy than the intermediate step. The experimental investigations suggest that the entropic barrier (54 kJ/mol) is non-negligible. In our simulations, this is expressed in the very narrow window of initial configurations that result in F attaching to PTA and subsequent bond breaking. Thus, the likelihood of the reaction taking place is largely determined by the unlikeliness of a specific initial configuration in addition to the energetic barrier that describes the bond breaking.

We estimated if our observations depend critically on the exchange-correlation potential in two ways. First, by calculating the Si-C C=C potential energy surface for the 60% configuration in ORCA using two different basis sets and two different functionals (see Supplementary Figure 3). While quantitative changes can be observed, they agree qualitatively such that our observations are likely to be robust against changing basis-set or DFT-functional.

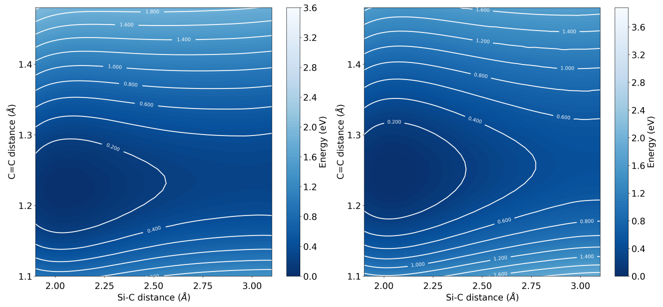

Supplementary Figure 3. Si-C C=C potential energy surface calculated with ORCA (F out of Si-C axis). Left, using the B3LYP functional and the def2-TZVP basis, right, using the PBE functional and the 6-31G* basis.

Second, by calculating in ORCA the vibrational spectrum and the corresponding Si-C stretching contribution for different levels of theoretical description, shown in Supplementary Figure 4. The positions of vibrational excitations and their Si-C stretching contribution is only marginally affected by the choice of functional, solvent or basis. Noticeable differences appear however in the relative spectral strength (especially around 300, 500 and 2000 cm^{-1}). In conclusion, our theoretical QEDFT calculations presented in the main text can be expected to provide a good description of the energetics and a qualitative description of the relevant mechanism. While our calculations suggest indeed a comparably small Si-C character in the experimentally coupled vibration, in agreement with [10], it seems as if the Si-C weighted spectrum provides a more reliable measure for the cavity influence on the reactivity. Supplementary Figure 4 indicates that especially the relative spectral strength is a sensitive quantity for which the theoretical calculations must be considered of limited reliability.

II. SUPPLEMENTARY DISCUSSION 1

We elaborated in the main text that coupling strongly to vibrations around $\omega_c = 570$ cm^{-1} leads to a particularly strong influence on the chemical reactivity. Supplementary Figure 5 illustrates the corresponding mode-projection, equivalent to fig. 3 in the main text. In com-

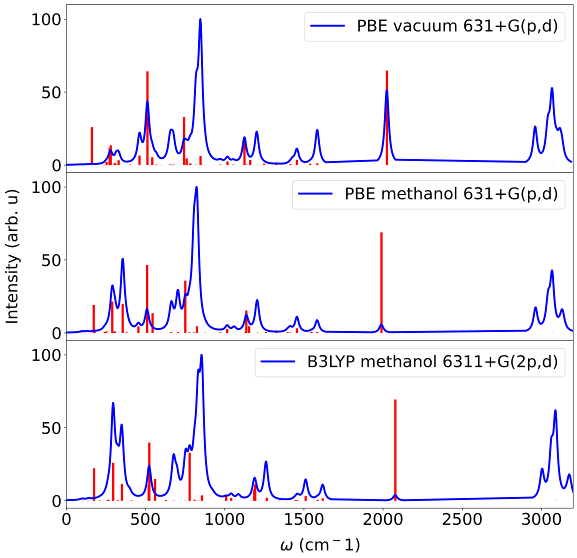

Supplementary Figure 4. Vibrational spectrum along a polarization aligned with the Si-C axis for different functionals, solvents and basis-sets. The spectra are obtained with ORCA using an artificial broadening of 30 cm^{-1} and the Si-C-stretching contributions are multiplied by 150 for better visibility. While energetic positions are largely insensitive to functional, solvent and basis, clear deviations in the relative spectral intensity become apparent. Our QEDFT calculations use a finite-difference grid, the PBE functional and vacuum.

parison with fig. 3, the vibration at 771 cm^{-1} is now less affected and the dominant effect of the resonant cavity is an accumulation of occupation around 500 cm^{-1}.

III. SUPPLEMENTARY DISCUSSION 2

Intuitively, energy redistribution between vibrations suggests (classical) correlation among them. If we assume that specific bonds are particularly characteristic for the involved vibrations, we might want to investigate to which extend those local bonds are correlated among each other. This would provide an intuitive understanding of the anharmonic couplings and local changes due to the cavity. Supplementary Figure 6 illustrates the classical bond correlation among a few most characteristic bonds in the F-Si-C=C-Benzene chain of PTAF^-. While minor changes in the correlation between Si-C and Si-F bond indicate that the F-Si-Meth complex undergoes vibrational changes depending on the resonant condition, those changes are small and do not allow to draw a clear conclusion.

IV. SUPPLEMENTARY DISCUSSION 3

Li et al. [11] suggested recently that the resonant effect of the slow down of a reaction under vibrational strong coupling originates from an effective solvent-caging effect around the transition-state. Thereby, the suggested resonant frequency emerges from the curvature of the PES at the transition-state $M\omega_b^2 = -\partial_R^2 E|_{TS}$. This would lead to a resonant frequency at $\omega = 74$ cm^{-1} [11]. We observe a clear resonance in the reactivity at $\omega = 86$ cm^{-1} which corresponds quite accurately to the transition-state curvature without solvent and the chosen PBE functional (Supplementary Table I). Furthermore, we obtain multiple additional resonances when the cavity is close to vibrations with substantial Si-C character, including the experimental resonance at 860 cm^{-1}. This suggests that both mechanisms, i.e., dynamical caging and vibrational energy redistribution, provide possible mechanism at the single-molecule strong-coupling level.

Supplementary Table I presents the binding frequency (lowest imaginary vibrational frequency at the transition-state) calculated with ORCA for different solvents, Gaussian basis-sets, and relative orientations of the F anion with respect to the Si-C bond-axis.

V. SUPPLEMENTARY DISCUSSION 4

As illustrated in Supplementary Figure 7, increasing light-matter coupling strength will lead to an increase of the cavity influence on the chemical reactivity. The shown trend is consistent with the rate-change measured in experiment (compare to fig. 3 a in [8], Rabi-splitting is $\propto g_0/\hbar\omega_c$). Although our single-particle coupling strength is considerably larger than in experiment, the cavity has to influence the reactive trajectory within a much shorter time in our calculations such that larger couplings are necessary for noticeable influence. The short reaction-time in our calculations originates from the selection of favourable initial states, representing a very small subset of the full statistical ensemble of all possible initial states.

VI. SUPPLEMENTARY DISCUSSION 5

When we increase the light-matter coupling strength, two distinct effects can be observed. First, the inhibition of the chemical reaction intensifies, that is, more trajectories will be stronger affected, second the approach of the F^- anion is delayed. Supplementary Figure 8 presents the forces acting on F projected on the cavity polarization axis for increasing light-matter coupling. During the dynamical approach of the F^- to the PTA complex, the methyl groups of the PTA complex need to rearrange to provide space for F^-. This leads to a short repellent force acting during the rearrangement time. The approaching F^- induces noticeable changes in the molecular dipole

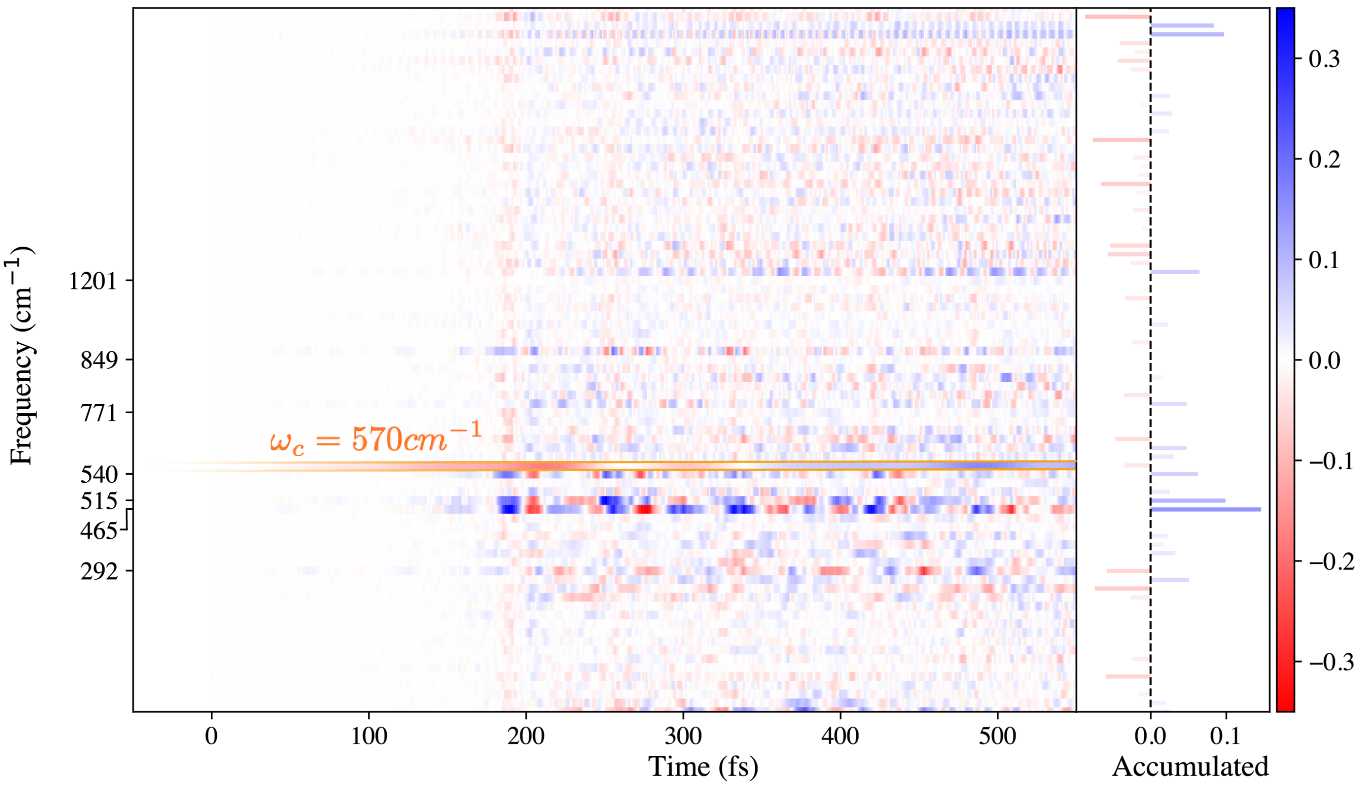

Supplementary Figure 5. Time-resolved influence on the mode occupations by resonant vibrational strong-coupling. Illustrated is the trajectory averaged difference in normalized mode-occupation between $\omega_c = 570$ cm^{-1}, $g_0/\hbar\omega_c = 1.132$ and far off-resonance $\omega_c = 43$ cm^{-1}, $g_0/\hbar\omega_c = 1.132$ for all 8 trajectories that undergo the reaction outside the cavity. The bar-plot shows the accumulated difference divided by a factor 100. The cavity mode (bordered orange) is here represented by the difference in normalized mode-displacement $q(t) = \sqrt{\hbar/2\omega_c}\langle\hat{a}^\dagger + \hat{a}\rangle$ and re-scaled by the factor $1/4$.

moment, resulting in an effective nuclear current coupling to the photonic system.

With increasing coupling strength, the latter results in an increasing repulsion which partially prevents F^- from attaching to PTA. The dynamic change of the molecular dipole with approaching F^- results therefore in an additional reaction-barrier. Considering that the reaction probability is slim in the first place, the success of F^- attacking and subsequently the rate of reactions is sensitive to any alternation of the F^- trajectory. This observation provides another possible explanation for the experimentally obtained large entropic change in the reaction character [8, 9] but would not feature the same dependence on the resonant condition as observed in the main paper and is comparably weak.

VII. SUPPLEMENTARY DISCUSSION 6

The phase space that leads to the reaction is very small, a major bottleneck is provides by the attachment of the F^- and the creation of the pentavalent complex. When the F^- attacks the complex, the methyl groups have to adjust their position and orientation, within the time-frame given by the approaching anion, in order to change from the tetra to a penta-structure. For this to realistically happen, the F^- needs to attack from the correct angle. After the successful creation of the pentavalent complex, the Si-C bond has to accumulate sufficient energy to break the bond. The low reactivity is nicely embodied by the possible angle of attack for the F^-. Our initial investigations found a second possible attack-angle at 60 degrees (compared to the Si-C axis) but the temperature necessary to see the reaction was more than 900 K, clearly not related to experiment. The preferential sampling is now inspired by Bayesian-like approaches where a higher sampling density is assigned to areas that are known to be more relevant for the reaction. Specifically, we sampled 20 trajectories with a relative deviation of 20K around a reactive trajectory. While this certainly does not exhaust the full reactive phase-space, it provides a good enough resolution to explore the basic mechanism of the reaction. This can be nicely seen from the Maxwell-Boltzmann distribution shown in Supplementary Figure 9.

Preferential sampling provides the methyl's with sufficient time and energy to re-arrange and facilitates the building process of the pentavalent complex. Adding more trajectories that are non-reactive would simply increase the weight of the reactant and thus lower the over-

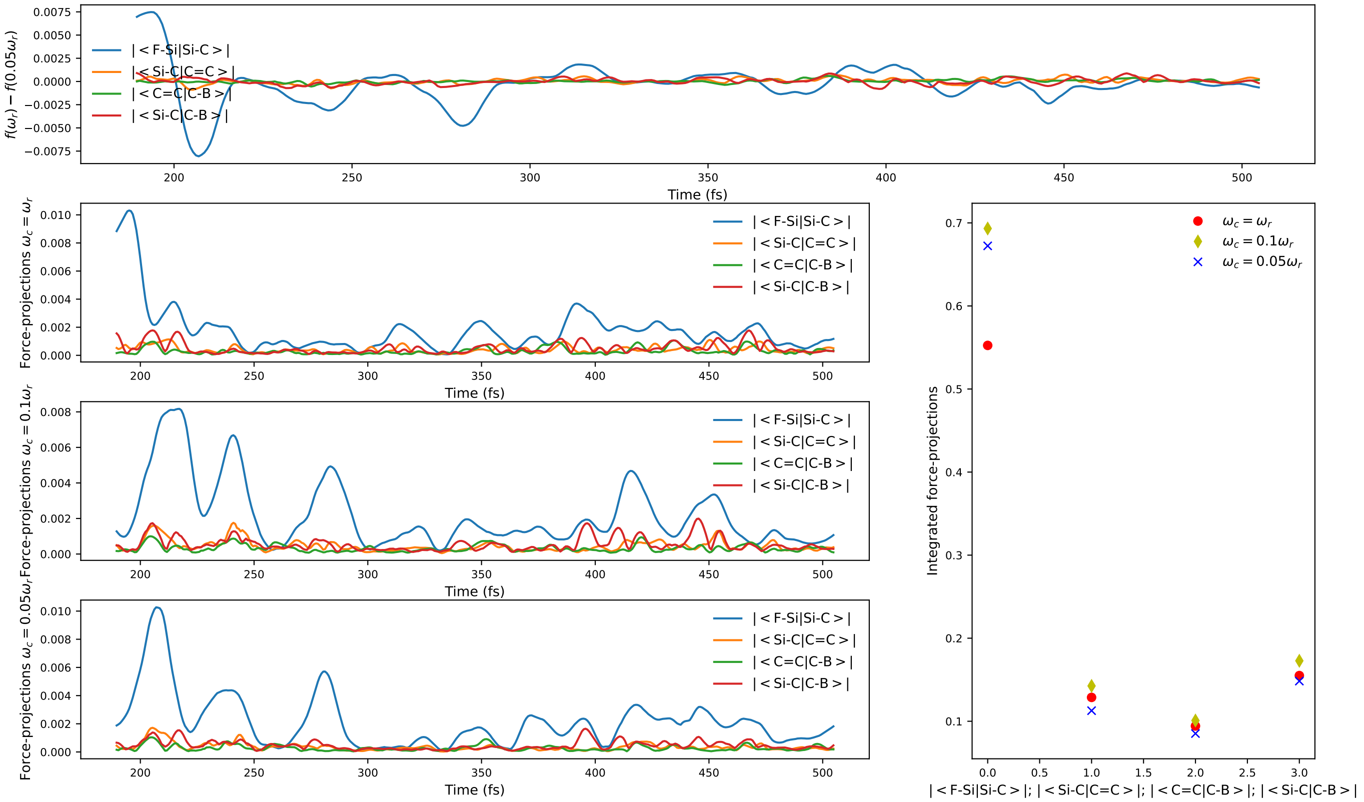

Supplementary Figure 6. Classical bond correlations for resonant ($\omega_r = 856$, 2nd row, left), lowest frequency (bottom, left), their difference (top), their integrated values (right) and the bond correlations for the dynamical caging resonance (3rd row, left). Calculated are the projections of the forces acting on the individual bonds, i.e., the classical time-dependent correlation between the forces acting on Si-C and Si-F bond (blue) are for instance $| < f_{Si} - f_C | f_{Si} - f_F > |$ which provides an estimate for how strong deformation of the Si-C bond influences deformation of the Si-F bond and vice versa. C-B denotes hereby the Carbon-Benzene bond and C=C the Carbon triple bond located between the Si-C functional group and the C-B bond. We see small differences in the Si-F Si-C bond correlations between the resonant and off-resonant situation. This might indicate that other bonds are substantially contributing in the cavity mediated energy redistribution (e.g. the Si-methyl bonds) but foremost clarifies that the Si-C bond is distributed over various vibrations. Future investigations could try to localize the vibrational modes (local vibrational mode basis) in order to better identify local changes that influence the Si-C bond breaking as a consequence of the resonant condition.

all average bond distance. We expect our conclusions to remain qualitatively accurate if the number of trajectories would be considerably increased.

VIII. SUPPLEMENTARY DISCUSSION 7

In this section, we briefly outline the computational method we are employing in this work. The method has been introduced earlier in Ref. [13] and we will stay here consistent with its naming conventions. We are working in the nonrelativistic limit, the length-gauge, and dipole approximation [14]. The Hamiltonian governing this limit of systems consisting of electrons, photons and nuclei has been introduced in the main text and is discussed in detail e.g. in Refs. [15, 16]. In Ref. [13] it has been demonstrated that a time-dependent (quantum-electrodynamical) density-functional theory can be constructed for general electron, nuclei and photon systems relying on the electron density n, the nuclear coordinates $\mathbf{R}_I$ and the photonic displacement coordinates q_α as basic variables. The equation of motion (EOM) for the photon coordinate is given by

$$\ddot{q}_\alpha(t) + \omega_\alpha^2 q_\alpha(t) + \omega_\alpha \boldsymbol{\lambda}_\alpha \cdot \boldsymbol{\mu}(t) = -j_{\mathrm{ext}}^{(\alpha)}(t)/\omega_\alpha. \quad (1)$$

which is a Maxwell's equations in the length-gauge with the external source term $-j_{\mathrm{ext}}^{(\alpha)}(t)/\omega_\alpha$. For the nuclear coordinates $\mathbf{R}_I$ We find

$$M_I \ddot{\mathbf{R}}_I(t) + \sum_{\beta=1}^{N_I} \sum_{\alpha=1}^{\mathcal{N}} Z_I \omega_\alpha \boldsymbol{\lambda}_\alpha \left(q_\alpha(t) + \frac{\boldsymbol{\lambda}_\alpha}{\omega_\alpha} \cdot \boldsymbol{\mu}(t) \right) + \sum_{\beta=1}^{N_I} \mathbf{F}_{\mathrm{str}}^{(I,\beta)}(t) = - \sum_{\beta=1}^{N_I} \mathbf{F}_{\mathrm{ext}}^{(I)}(t), \quad (2)$$

where we have used the nuclear stress force $\mathbf{F}_{\mathrm{str}}^{(I,\beta)}(t) = \langle \Psi(t) | \vec{\nabla}_{I,\beta} \hat{V}(\underline{\mathbf{r}}, \underline{\mathbf{R}}) | \Psi(t) \rangle$ and an external forces on the nuclei $\mathbf{F}_{\mathrm{ext}}^{(I)}(t)$. The electronic system is then propagated

Theory level	Solvent	F position	ω_b [cm^{-1}]
PBE 6-31G*	Vacuum	in-axis	90
PBE 6-31G*	Methanol	in-axis	94
PBE 6-311G*	Methanol	in-axis	88
B3LYP 6-31G*	Methanol	in-axis	93
PBE 6-31G*	Acetonitrile	in-axis	94
PBE 6-31G*	Vacuum	off-axis	85
PBE 6-311G*	Methanol	off-axis	81
Li et al. [11]	Methanol	in-axis	74
			Resonance [cm^{-1}]
QEDFT (PBE)	Vacuum	in-axis	$\approx$ 86
			$\approx$ 292
			$\approx$ 515
			$\approx$ 771
			$\approx$ 849
			$\approx$ 1201
Experiment [8]	Methanol	-	860
Related exp. [12]	Methanol	-	1250

Supplementary Table I. Lowest (imaginary) vibrational frequency at the transition-state for the two different configurations calculated with different solvents and theoretical descriptions. The QEDFT calculations capture the dynamical caging effect around the binding frequency in addition to the other resonances at vibrational frequencies with strong Si-C character. We decided to label the QEDFT resonances by the associated vibrational excitations, our time-dependent calculations suggest an increasing blue-shift for increasing frequency. The available experiments [8, 9] suggests a resonance at 860 cm^{-1} and a related experiment at 1250 cm^{-1} [12].

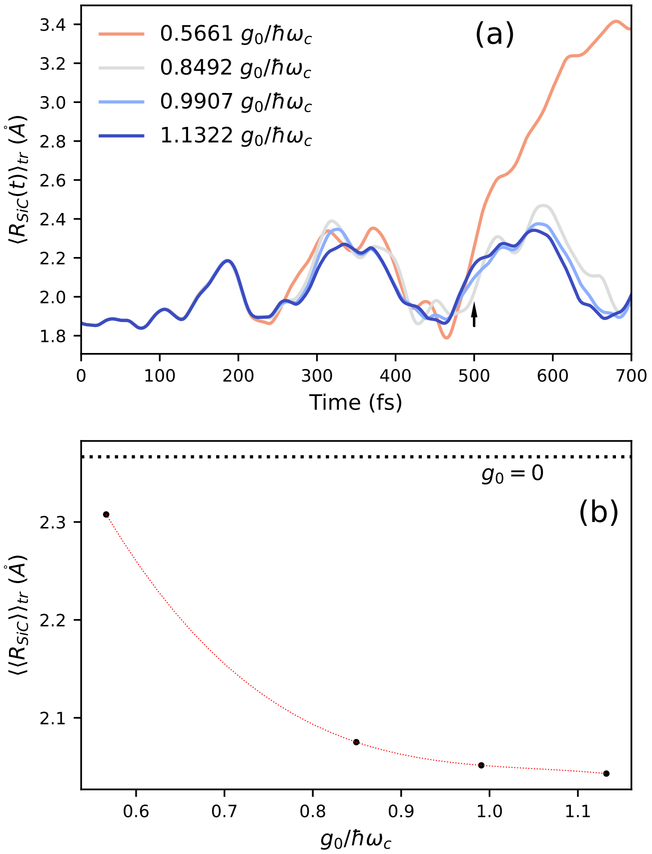

Supplementary Figure 7. Time-resolved trajectory averaged Si-C distance (a) and time-averaged Si-C distance $\langle\langle R_{SiC}\rangle\rangle_{tr}$ (b) for cavity frequency $\omega_c = 571$ cm^{-1}. The ratio $g_0/\hbar\omega_c$ is varied, the red-dotted line represents a cubic spline interpolation and serves as guide to the eye. We use all 8 trajectories that show the reaction outside the cavity.

in the Kohn-Sham system

$$i\frac{\partial}{\partial t}\varphi_i(\mathbf{r},t) = \left[-\frac{\vec{\nabla}_i^2}{2} + v_s(\mathbf{r},t)\right]\varphi_i(\mathbf{r},t) \tag{3}$$

with the following Kohn-Sham potentials

$$v_s(\mathbf{r},t) = v_{\text{ext}}(\mathbf{r},t) + v_{\text{Mxc}}(\mathbf{r},t) \tag{4}$$

with the mean-field xc potential

$$v_{\text{Mxc}}(\mathbf{r},t) = v_{\text{Hxc}}(\mathbf{r},t) + \sum_{\alpha=1}^{\mathcal{N}} v_{\text{Mxc}}^{(\alpha)}(\mathbf{r},t) + v_{\text{Mxc}}^{(N)}(\mathbf{r},t), \tag{5}$$

In this approach, we approximate the matter-light dynamics, and the electron-nuclear by a semiclassical approximation [13]. The electron-nuclear dynamics becomes identical to Ehrenfest dynamics [17].

IX. SUPPLEMENTARY DISCUSSION 8

Thomas et al. [8] found a Full Width at Half Maximum (FWHM) of the cavity eigenmode of 30 cm^{-1} which corresponds via $\tau_{cav} = 1/\Delta f = 1/(\tilde{\nu}c) = 1/(15cm^{-1}2.998 \cdot 10^{10}cm/s)$ to a lifetime of 2.22 ps – about 4 times the time of the reaction. This would suggest a rather small influence on our reactive trajectories. Nevertheless, this section investigates the influence of photonic loss on the effect of the cavity. Some previous studies [18] suggest that larger losses amplify the strength of cavity mediated effects while other investigations see little influence with the tendency to hinder cavity mediated effects [19]. Losses compete with the onset of strong coupling and all available experimental results indicate no relevant effect of the cavity if the strong coupling domain is not reached. The electromagnetic displacement field follows the mode projected Maxwell equation [15].The imperfection of the cavity can be conveniently included via a friction term

$$\partial_t^2 q_{\mathbf{k}\lambda}(t) + 2\gamma\partial_t q_{\mathbf{k}\lambda}(t) + \omega_{\mathbf{k}\lambda}^2 q_{\mathbf{k}\lambda}(t) = \omega_{\mathbf{k}\lambda}\boldsymbol{\lambda}_{\mathbf{k}\lambda}\cdot\mathbf{R}(t)\ . \tag{6}$$

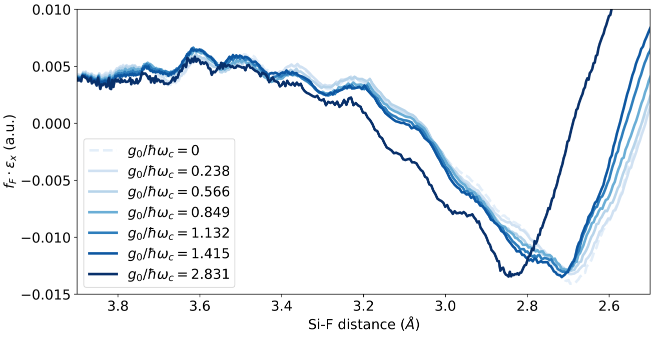

Supplementary Figure 8. Force acting on the F atom along the cavity polarization axis for the to fig. 2 (resonant) corresponding trajectory. With increasing light-matter coupling a weak repellent force is induced that prevents the F anion to attack the PTA complex. The Ehrenfest light-matter coupling utilized here becomes less reliable for larger coupling strength, the overall strength of the effect can be expected to be weak for experimental values.

The slightly modified Maxwell equation is solved by the Greens function

$$G_{\mathbf{k}\lambda}(t,t') = \frac{\left(e^{[\sqrt{\gamma^2-\omega_{\mathbf{k}\lambda}^2}-\gamma](t-t')} - e^{-[\sqrt{\gamma^2-\omega_{\mathbf{k}\lambda}^2}+\gamma](t-t')}\right)}{2\sqrt{\gamma^2-\omega_{\mathbf{k}\lambda}^2}} \tag{7}$$

where $\pm\sqrt{\gamma^2-\omega_{\mathbf{k}\lambda}^2}-\gamma$ are the roots of the quadratic equation $x^2+2\gamma x+\omega^2=0$. This leads to a damped oscillation of the strongly-coupled mode.

The full description for the mode q considering the homogeneous solution and the initial state problem can be deduced from the ansatz $q^{hom}(t) = AG(t,t_0) + AG(t+\phi, t_0-\phi)$. Ignoring the possibility for initial momenta $\dot{q}_0 = 0$, we obtain with

$$\begin{aligned} q_{\mathbf{k}\lambda}(t) &= \int_{t_0}^{t} dt' G_{\mathbf{k}\lambda}(t,t')\omega_{\mathbf{k}\lambda}\boldsymbol{\lambda}_{\mathbf{k}\lambda}\cdot\mathbf{R}(t') \\ &\quad - \frac{i}{2}q_{0,\mathbf{k}\lambda}\tilde{G}_{\mathbf{k}\lambda}(t,t_0) \\ \tilde{G}_{\mathbf{k}\lambda}(t,t_0) &= e^{[\sqrt{\gamma^2-\omega_{\mathbf{k}\lambda}^2}-\gamma](t-t_0)+i\frac{\pi}{2}} \\ &\quad - e^{-[\sqrt{\gamma^2-\omega_{\mathbf{k}\lambda}^2}+\gamma](t-t_0)-i\frac{\pi}{2}} \end{aligned} \tag{8}$$

the 'lossy cavity' Maxwell potential

$$v_M(\mathbf{r}t) = \sum_{\mathbf{k}\lambda}\boldsymbol{\lambda}_{\mathbf{k}\lambda}\cdot\hat{\mathbf{R}}\big[\omega_{\mathbf{k}\lambda}q_{\mathbf{k}\lambda}(t) - \boldsymbol{\lambda}_{\mathbf{k}\lambda}\cdot\mathbf{R}(t)\big] \tag{9}$$

with total dipole moment $\hat{\mathbf{R}} = \sum_{i=1}^{N_n} Z_i\hat{\mathbf{R}}_{n,i} - e\sum_{i=1}^{N_e}\hat{\mathbf{r}}_i$. The new potential has been implemented into Octopus and tested against against a small Python library.

Supplementary Figure 10 shows only minor influence on the suppression of chemical reactivity with $\omega_c = 571$ cm^{-1} for the experimentally observed ($2\gamma_{exp} = 0.000137$ a.u.) or amplified ($\gamma = \{0,1,2,4,8\}\cdot\gamma_{exp}$) losses.

X. SUPPLEMENTARY DISCUSSION 9

The majority of the trajectories undergoing the reaction far off-resonant or in free-space no longer show a reactive behaviour close to any of the discussed resonances. For all of those cases, the trajectory is trapped close to the local minimum at the pentavalent PTAF complex, potentially stretching the Si-C bond but relaxing back to the local minimum. Single trajectories can however undergo the reaction at longer times as Supplementary Figure 11 illustrates. As the statistical ensemble becomes increasingly insufficient the longer the time-propagation, we decide to limit the presentation of the resonant effect to the first 700 fs. If we would select a much smaller coupling strength, the influence of the cavity would be also substantially smaller, i.e., many of the aforementioned trajectories close to resonance would resemble closer those off-resonance. One should note hereby that the reaction is unlikely to happen from the start, our ensemble is already sampled around the ideal initial state which leads to the quick reaction speed of ps compared to the minutes of acquisition in experiment. This implies, that a much larger ensemble and a much longer propagation time would be needed to resolve the reaction and its changes if we would not select such a strong light-matter strength and preferable initial state. Consequently, the present computational limitations advice the here presented approach.

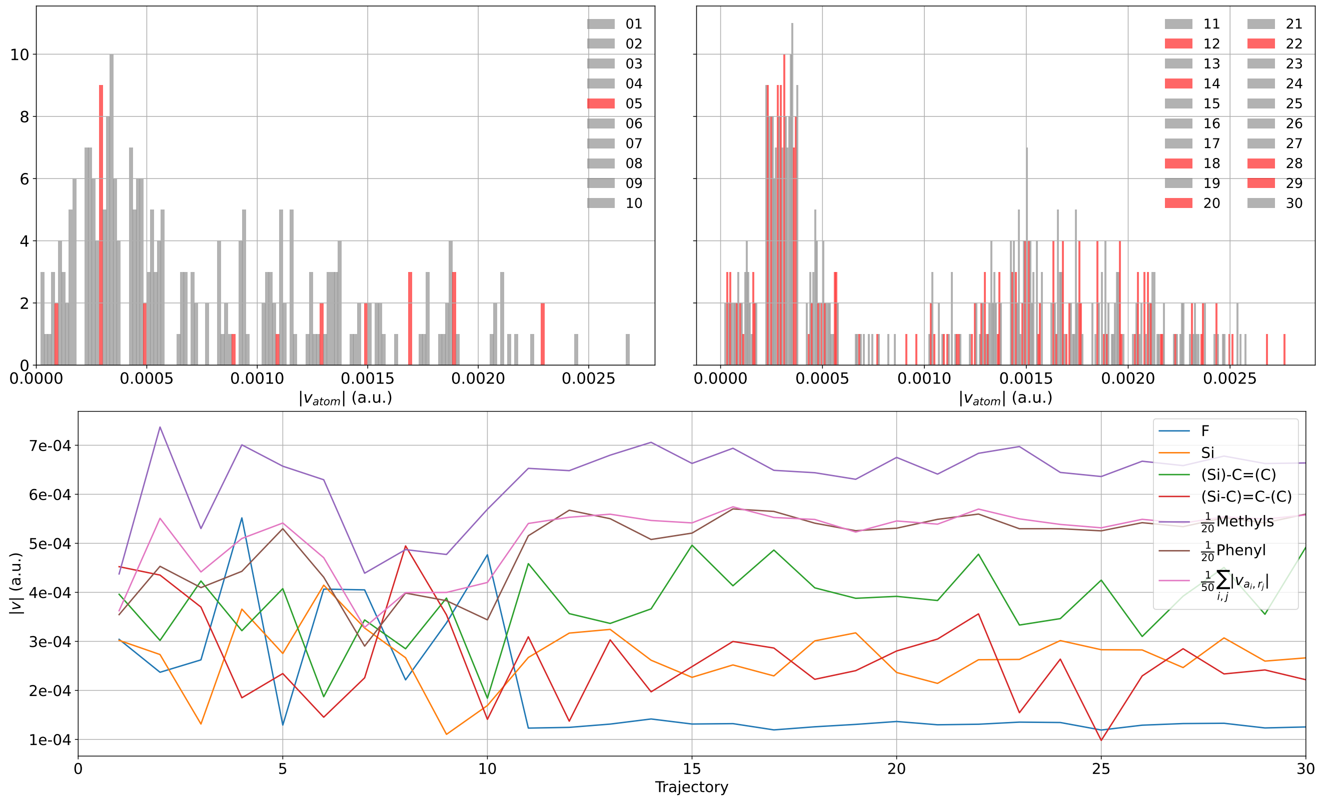

Supplementary Figure 9. (Top) Maxwell-Boltzmann histogram, counting how many atoms lie within a given velocity-range, for the investigated set of trajectories, reactive in red. A pronounced high-energy tail is necessary to undergo reaction, our preferential sampling increases the probability for such a trajectory to be used within the calculation. It should be noted that even non-reactive trajectories can exhibit strong Si-C bond-stretchings which contributes additional information to our analysis in Fig. 4.
(Bottom) Densely sampling of the high-energy tail corresponds to an in average higher temperature of the methyl groups while the attacking F^- is slower.

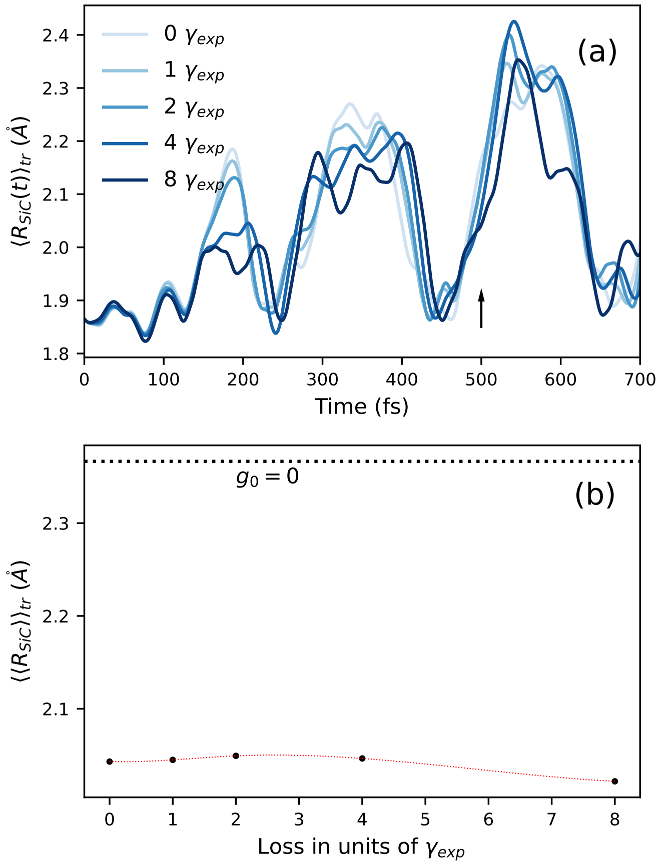

Supplementary Figure 10. Time-resolved trajectory averaged Si-C distance (a) and time-averaged Si-C distance $\langle\langle R_{SiC}\rangle\rangle_{tr}$ (b) for cavity frequency $\omega_c = 571$ cm^{-1} and $g_0/\hbar\omega_c = 1.1322$ with increasing cavity loss γ. The red-dotted line represents a cubic spline interpolation and serves as guide to the eye. We use all 8 trajectories that show the reaction outside the cavity.

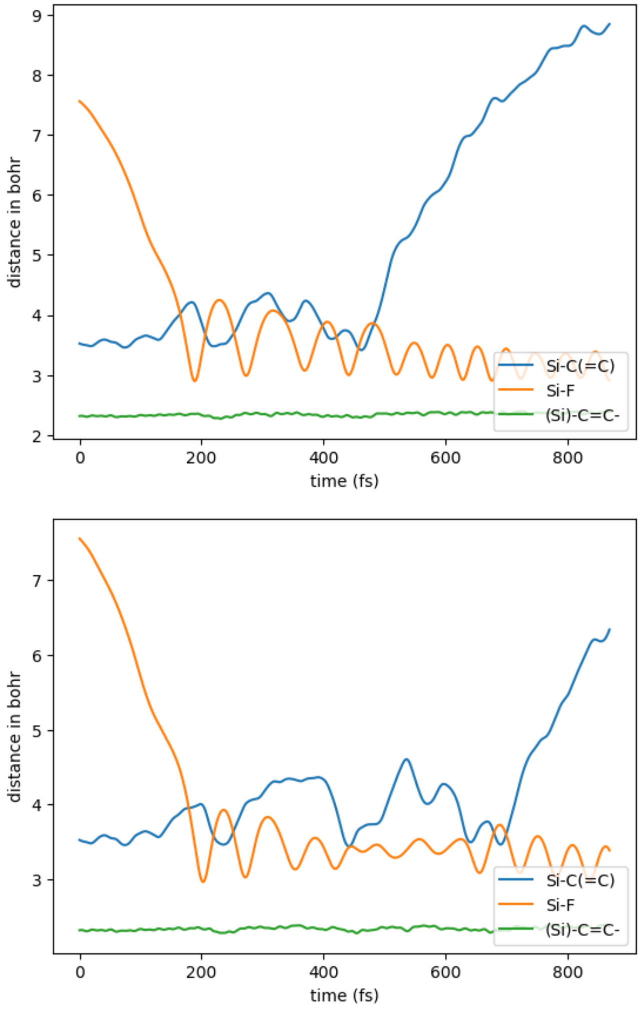

Supplementary Figure 11. Time-dependent bond distances in free-space (top) and at $\omega_c = 86$ cm^{-1} (bottom). The Si-C bond (blue) crosses the transition-state at around 500 fs in free-space and at 800 fs around the dynamical caging frequency.

XI. SUPPLEMENTARY REFERENCES

[1] J. Bonini and J. Flick, Ab initio linear-response approach to vibro-polaritons in the cavity born–oppenheimer approximation, Journal of Chemical Theory and Computation **18**, 2764 (2022), pMID: 35404591, https://doi.org/10.1021/acs.jctc.1c01035.

[2] C. Schäfer, M. Ruggenthaler, H. Appel, and A. Rubio, Modification of excitation and charge transfer in cavity quantum-electrodynamical chemistry, Proceedings of the National Academy of Sciences **116**, 4883 (2019).

[3] C. Schäfer, F. Buchholz, M. Penz, M. Ruggenthaler, and A. Rubio, Making ab initio qed functional (s): Nonperturbative and photon-free effective frameworks for strong light–matter coupling, Proceedings of the National Academy of Sciences **118** (2021).

[4] J. Flick, M. Ruggenthaler, H. Appel, and A. Rubio, Atoms and molecules in cavities, from weak to strong coupling in quantum-electrodynamics (qed) chemistry, Proceedings of the National Academy of Sciences **114**, 3026 (2017).

[5] J. Flick, C. Schäfer, M. Ruggenthaler, H. Appel, and A. Rubio, Ab initio optimized effective potentials for real molecules in optical cavities: Photon contributions to the molecular ground state, ACS Photonics **5**, 992 (2018), https://doi.org/10.1021/acsphotonics.7b01279.

[6] T. S. Haugland, E. Ronca, E. F. Kjønstad, A. Rubio, and H. Koch, Coupled cluster theory for molecular polaritons: Changing ground and excited states, Physical Review X **10**, 041043 (2020).

[7] T. S. Haugland, C. Schäfer, E. Ronca, A. Rubio, and H. Koch, Intermolecular interactions in optical cavities: An ab initio qed study, The Journal of Chemical Physics **154**, 094113 (2021).

[8] A. Thomas, J. George, A. Shalabney, M. Dryzhakov, S. J. Varma, J. Moran, T. Chervy, X. Zhong, E. Devaux, C. Genet, J. A. Hutchison, and T. W. Ebbesen, Ground-state chemical reactivity under vibrational coupling to the vacuum electromagnetic field, Angewandte Chemie International Edition **55**, 11462 (2016).

[9] A. Thomas, A. Jayachandran, L. Lethuillier-Karl, R. M. Vergauwe, K. Nagarajan, E. Devaux, C. Genet, J. Moran, and T. W. Ebbesen, Ground state chemistry under vibrational strong coupling: dependence of thermodynamic parameters on the rabi splitting energy, Nanophotonics **9**, 249 (2020).

[10] C. Climent and J. Feist, On the SN2 reactions modified in vibrational strong coupling experiments: reaction mechanisms and vibrational mode assignments, Physical Chemistry Chemical Physics **22**, 23545 (2020).

[11] X. Li, A. Mandal, and P. Huo, Cavity frequency-dependent theory for vibrational polariton chemistry, Nature Communications **12**, 10.1038/s41467-021-21610-9 (2021).

[12] A. Thomas, L. Lethuillier-Karl, K. Nagarajan, R. M. A. Vergauwe, J. George, T. Chervy, A. Shalabney, E. Devaux, C. Genet, J. Moran, and T. W. Ebbesen, Tilting a ground-state reactivity landscape by vibrational strong coupling, Science **363**, 615 (2019).

[13] J. Flick and P. Narang, Cavity-correlated electron-nuclear dynamics from first principles, Phys. Rev. Lett. **121**, 113002 (2018).

[14] D. Craig and T. Thirunamachandran, *Molecular Quantum Electrodynamics: An Introduction to Radiation-molecule Interactions*, Dover Books on Chemistry Series (Dover Publications, 1998).

[15] I. V. Tokatly, Time-dependent density functional theory for many-electron systems interacting with cavity photons, Phys. Rev. Lett. **110**, 233001 (2013).

[16] M. Ruggenthaler, J. Flick, C. Pellegrini, H. Appel, I. V. Tokatly, and A. Rubio, Quantum-electrodynamical density-functional theory: Bridging quantum optics and electronic-structure theory, Phys. Rev. A **90**, 012508 (2014).

[17] X. Andrade, A. Castro, D. Zueco, J. Alonso, P. Echenique, F. Falceto, and A. Rubio, Modified ehrenfest formalism for efficient large-scale ab initio molecular dynamics, Journal of chemical theory and computation **5**, 728 (2009).

[18] A. Mandal, X. Li, and P. Huo, Theory of vibrational polariton chemistry in the collective coupling regime, The Journal of Chemical Physics **156**, 014101 (2022).

[19] C. Schäfer, Polaritonic chemistry from first principles via embedding radiation reaction, The Journal of Physical Chemistry Letters **13**, 6905 (2022), pMID: 35866694, https://doi.org/10.1021/acs.jpclett.2c01169.
